# Farrerol inhibits ferroptosis and protects against LPS-induced acute lung injury by targeting the RUNX1/SLC7A11 axis

**DOI:** 10.3389/fimmu.2025.1720843

**Published:** 2026-01-07

**Authors:** Yueyan Yang, Shihua Deng, Teng Liu, Qing Yin, Min Yang, Xintao Zhang, Zhongyong Jiang, Xiaobian Wang, Sunhan Zhang, Ting Zhang, Dongming Wu, Ying Xu

**Affiliations:** 1 School of Clinical Medicine, Chengdu Medical College, Chengdu, Sichuan, China; 2 The First Affiliated Hospital of Chengdu Medical College, Chengdu, Sichuan, China; 3 Department of Medical Laboratory, Affiliated Cancer Hospital of Chengdu Medical College, Chengdu Seventh People’s Hospital, Chengdu, Sichuan, China; 4 Sichuan Clinical Research Center for Radiation and Therapy, The First Affiliated Hospital of Chengdu Medical College, Chengdu, Sichuan, China; 5 Clinical In Vitro Diagnostic (IVD) Joint Research Center of Chengdu Medical College-Maccura Biotechnology, Chengdu, Sichuan, China

**Keywords:** acute lung injury, farrerol, ferroptosis, RUNX1, SLC7A11

## Abstract

**Introduction:**

Acute lung injury (ALI) is a critical condition with diverse etiologies, characterized by high mortality rates and a lack of specific therapeutic interventions. Farrerol, a naturally occurring flavonoid isolated from *Rhododendron* spp., exhibits potent anti-inflammatory and antioxidant activities. Previous studies have indicated that farrerol exerts protective effects against lipopolysaccharide (LPS)-induced ALI; however, the underlying mechanisms remain elusive. This study aimed to elucidate the protective mechanisms of farrerol against LPS-induced ALI.

**Methods:**

We evaluated the efficacy of farrerol using both *in vitro* (LPS-stimulated BEAS-2B cells) and *in vivo* (LPS-induced ALI in mice) models. The protective mechanism of rhododendron against ALI was investigated using proteomics, cellular thermal shift assays, co-immunoprecipitation, and molecular docking.

**Results:**

Pretreatment with farrerol significantly improved cell viability and reduced lactate dehydrogenase release in LPS-induced BEAS-2B cells. *In vivo*, farrerol effectively alleviated LPS−induced pulmonary edema and histopathological damage in mice. Mechanistically, we found that farrerol directly binds to and stabilizes runt-related transcription factor 1 (RUNX1), thereby transcriptionally activating the expression of solute carrier family 7 member 11. Overexpression of RUNX1 mimicked the protective effects of farrerol, while knockdown of RUNX1 abolished these effects.

**Discussion:**

Farrerol could directly bind and stabilize the expression of RUNX1, thereby enhancing *SLC7A11* transcription and ultimately inhibiting ferroptosis. Thus, farrerol is a potential therapeutic agent for ALI.

## 1 Introduction

Acute lung injury (ALI) is a life-threatening pulmonary disorder triggered by diverse etiological factors, including infections, trauma, and toxic inhalants. It is characterized by disruption of the alveolar-capillary barrier, oxidative stress imbalance, and impaired gas exchange, with severe cases progressing to Acute Respiratory Distress Syndrome (1, 2). Mounting evidence indicates that the pathogenesis of ALI is closely associated with lipid peroxidation, aberrant reactive oxygen species (ROS) accumulation, and dysregulated iron homeostasis (3–5). Despite advancements in clinical management, current therapeutic strategies remain predominantly supportive owing to the incomplete elucidation of their molecular regulatory networks. This underscores the urgent need to dissect oxidative injury mechanisms and identify novel therapeutic targets for ALI (6).

LPS, a classic cause of sepsis, has been widely used to establish animal models of ALI (7). LPS induces excessive ROS generation through activation of the nicotinamide adenine dinucleotide phosphate oxidase system, leading to mitochondrial dysfunction and a cascade of lipid peroxidation in pulmonary tissues (8). Notably, ferroptosis—an iron-dependent, lipid peroxidation-driven form of programmed cell death—is significantly associated with the pathological progression of ALI (9). The characteristic hallmarks of ferroptosis include inhibition of glutathione peroxidase 4 (GPX4) activity, downregulation of solute carrier family 7 member 11 (*SLC7A11*) expression, and accumulation of lipid peroxidation end-products. These molecular events may exacerbate tissue damage by compromising the integrity of pulmonary epithelial cells (10–12). However, the dynamic regulatory network of ferroptosis in ALI and its critical molecular switches remain to be systematically elucidated.

Flavonoids have garnered extensive attention owing to their multi-target regulatory properties and low toxicity profile (13). Several studies have demonstrated their pulmonary protective effects through mechanisms such as free radical scavenging, iron metabolism modulation, and inhibition of lipid peroxidation (14, 15). Farrerol, a flavonoid derived from the traditional Chinese medicinal plant *Rhododendron dauricum*, has demonstrated unique therapeutic potential because of its potent antioxidant activity and ability to regulate cell death pathways (16–18). Studies have confirmed that farrerol effectively mitigates oxidative stress-related tissue damage by enhancing cellular antioxidant defenses and regulating iron metabolism-related proteins (19, 20). Importantly, farrerol attenuates ferroptosis by suppressing lipid peroxidation and modulating iron metabolism (21). However, the precise molecular mechanisms underlying the effects of farrerol on ALI, particularly its role in regulating ferroptosis within alveolar epithelial cells, remain elusive (22, 23).

Therefore, we addressed this gap by employing comprehensive *in vivo* and *in vitro* ALI models to determine whether farrerol exerts a protective effect against LPS-induced ALI. In addition, through a series of molecular and functional experiments, we investigated the direct molecular targets and downstream signaling pathways of farrerol. Finally, after confirming that farrerol can activate *SLC7A11* through its direct target runt-related transcription factor 1 (RUNX1), we examined whether RUNX1/*SLC7A11*-mediated ferroptosis is involved in the protective effects exerted by farreol.

## 2 Materials and methods

### 2.1 Reagents

The reagents used in this study include Farrerol (S9552), procured from Selleckchem; Lipopolysaccharide (LPS, catalogue number L2880), sourced from Sigma Aldrich Co., Ltd (US). RUNX1 (25315-1-AP, 1:2000 for WB,1:200 for IF), GAPDH (60004-1-Ig, 1:5000 for WB), EIF3E (10899-1-AP, 1:2000 for WB), SLC25A24 (14669-1-AP, 1:2000 for WB), BAX (50599-2-Ig, 1:3000 for WB), BCL-2 (26593-1-AP, 1:3000 for WB), RIPK3 (29080-1-AP, 1:2000 for WB), GPX4 (67763-1-Ig, 1:2000 for WB), Ubiquitin Antibody (91257, 1:2000 for WB), HRP-labeled Goat Anti-Rabbit IgG (H+L) (SA00001-2, 1:8000 for WB), HRP-labeled Goat Anti-Mouse IgG (H+L) (SA00001-1, 1:8000 for WB), all were purchased from Proteintech (Wuhan, China). SLC7A11 (ab307601, 1:1500 for WB, 1:300 for IF), GSDMD (ab209845, 1:1500 for WB), 4-Hydroxynonenal (4-HNE) (ab48506, 1:1500 for WB), were bought from Abcam (Cambridge, UK). Cy3-labeled Goat Anti-Rabbit IgG (H+L) (A0516, 1:200 for IF), Cy3-labeled Goat Anti-Mouse IgG (H+L) (A0521, 1:200 for IF), FITC-labeled Goat Anti-Rabbit IgG (H+L) (A0562, 1:200 for IF), FITC-labeled Goat Anti-Mouse IgG (H+L) (A0568, 1:200 for IF), Alexa Fluor 488-labeled Goat Anti-Rabbit IgG (H+L) (A0423, 1:200 for IF), Alexa Fluor 488-labeled Goat Anti-Mouse IgG (H+L) (A0428, 1:200 for IF), BeyoMag™ Mouse IgG Magnetic Beads (P2171-1ml), and BeyoMag™ Rabbit IgG Magnetic Beads (P2173-1ml), all were supplied by Beyotime Biotechnology Co., Ltd (Shanghai, China). Caspase-1 (D7F10, 1:2000 for WB), MLKL (14993, 1:2000 for WB), Phospho-MLKL (91689, 1:2000 for WB), Rabbit Anti-Biotin/PE-Cy3 (Acmec, AC55140, 1:2000), purchased from Cell Signaling Technology (Danvers, USA) and Acmec (Shanghai, China). DAPI (4′,6-Diamidino-2-phenylindole) (C0065, 1:5000 for WB), purchased from Solarbio Co., Ltd (Beijing, China).

### 2.2 *In vitro* experiments

#### 2.2.1 Cell culture and transfection

BEAS-2B cells were cultured in RPMI-1640 medium containing 10% FBS, with the medium purchased from Hyclone (Hudson, NH, USA). The cells were maintained in an incubator at 37 °C in an atmosphere containing 5% CO_2_ (Thermo, US). In accordance with the manufacturer’s guidelines, siRNA transfection was performed using Lipofectamine 3000 (Invitrogen, Thermo Fisher Scientific) at a final concentration of 20 nM. Post-transfection, the cells were incubated for 36 hours under the aforementioned conditions. Subsequently, the transfection medium was replaced with a fresh, complete medium in preparation for further experimental analysis. The siRNA sequences targeting RUNX1 used in this study are as follows: RUNX1-1: 5¢-GCUGAGCUGAGAAAUGCUAdTdT-3¢ (human); RUNX1-2: 5¢-CCUCGAAGACAUCGGCAGAAAdTdT-3¢ (human); RUNX1-3: 5¢-GAACCACUCCACUGCCUUUAAdTdT-3¢ (human).

#### 2.2.2 Establishment of BEAS-2B cell injury model

An LPS-induced injury model in BEAS-2B cells was established to evaluate the protective effect of Farrerol *in vitro*. Cells were seeded at a density of 5 × 10³ cells/well in 96-well plates or 1 × 10^5^ cells/well in 6-well plates and cultured at 37 °C in a humidified incubator with 5% CO_2_ until reaching 70–80% confluence. Cells were then treated with LPS at concentrations of 2.5, 5, 10, and 20 μg/mL for 6, 12, or 24 hours. Samples were collected at each time point to determine the optimal conditions (dose/time) for inducing injury. All procedures were performed under sterile conditions, with regular medium changes. A blank control group was included to assess LPS-induced inflammatory effects and the protective efficacy of the compound.

#### 2.2.3 Overexpression plasmid construction and transfection

A RUNX1 overexpression plasmid containing human RUNX1 cDNA was constructed using the PGMLV-6395 vector backbone (synthesized by Genomeditech) and verified by PCR and Sanger sequencing. BEAS-2B cells were transfected with 2 µg/mL plasmid using Lipofectamine 3000 (Thermo Fisher Scientific) according to the manufacturer’s instructions. Cells were harvested 48 hours post-transfection for analysis of RUNX1 mRNA and protein expression to confirm successful overexpression.

#### 2.2.4 Cell viability assay (CCK-8), cytotoxicity assay (LDH release), Cell proliferation assay (EdU), intracellular ROS measurement (DCFH-DA) and mitochondrial membrane potential assay

Cell viability, cytotoxicity, proliferation, intracellular ROS levels were assessed using CCK-8, LDH, EdU, and ROS assay kits (Beyotime, Shanghai, China), while MMP in BEAS-2B cells was evaluated using the JC-1 Mitochondrial Membrane Potential Detection Kit (Biotium, #C2003S). All assays were executed following the manufacturer’s standardized protocols.

#### 2.2.5 FerroOrange assay

BEAS-2B cells were cultured with a density of 1×104 cells/well for a day and stimulated with corresponding drugs for another day. FerroOrange probe (1 μM) was used to detect Fe^2+^ (Dojindo Laboratories, #F374). Iron quantification in lung tissue: Lung iron content was determined using a colorimetric assay kit (Abcam, #ab83366) according to the manufacturer’s protocol.

#### 2.2.6 Flow cytometry 7-AAD staining, immunofluorescence staining of cells and western blotting

Cellular death was quantified via 7-AAD staining and flow cytometry, and protein-protein interactions between Far and RUNX1 were analyzed by confocal immunofluorescence co-localization. Western blotting assays were performed to measure the expressions of RUNX1, EIF3E, SLC25A24, Bax, Bcl2, RIPK3, p-MLKL, Caspase-1, GSDMD-N, GPX4, 4-HNE. All experimental procedures were performed following our previously established protocols (5, 12).

#### 2.2.7 Ferroptosis PCR array analysis

A ferroptosis-related gene expression profile regulated by RUNX1 was analyzed using a ferroptosis PCR array (wc-mRNA0271-H, Woji Biotech). Total RNA was extracted from BEAS-2B cells using the Solarbio RNA extraction kit (R1200), and RNA quality was assessed by NanoDrop spectrophotometry and agarose gel electrophoresis. Reverse transcription was performed using the iScript cDNA Synthesis Kit (Bio-Rad, 1708890), and qPCR was carried out on a CFX96 system using SYBR Green Supermix (Bio-Rad, 1708880). Each sample was analyzed in triplicate. Data were processed using the 2^(-ΔΔCt) method via the Woji Biotech analysis platform. All procedures were conducted under standardized conditions to ensure reproducibility.

#### 2.2.8 Pull-down experiment

A pull-down assay was performed using biotin-labeled Farrerol (20 µM) to identify binding targets in BEAS-2B cell lysates. Cells were lysed with RIPA buffer, and the supernatant was collected by centrifugation. Lysates were incubated overnight at 4 °C with biotin-Farrerol, followed by incubation with streptavidin-conjugated magnetic beads for 2 hours at 4 °C to capture target proteins. After three washes with pre-chilled PBS, bound proteins were separated by SDS-PAGE and visualized by Coomassie Brilliant Blue staining. Specific bands were excised and analyzed by mass spectrometry to identify potential binding targets.

#### 2.2.9 Cell thermal shift assay

Cellular Thermal Shift Assay (CETSA) was performed to verify the direct interaction between Farrerol and its target protein. BEAS-2B cells were treated with a biotin-labeled Farrerol probe (prepared by Xi’an Qiyue Biotechnology Co., Ltd.) at 37 °C for 1 hour, followed by heat treatment at a temperature gradient (48–73 °C) for 10 minutes. After rapid cooling on ice, cells were lysed with RIPA buffer containing protease inhibitors. Lysates were centrifuged at 12,000 rpm for 15 minutes, and supernatants were analyzed by Western blotting.

#### 2.2.10 Dual-luciferase reporter assay

BEAS-2B cells were co-transfected with the pGL3-SLC7A11 promoter reporter plasmid and a Renilla luciferase internal control plasmid, with or without RUNX1 overexpression. After 48 hours, luciferase activity was measured using the GM-040503B dual-luciferase assay kit. The Firefly/Renilla ratio was calculated to quantify the transcriptional activation effect of RUNX1 on the SLC7A11 promoter.

#### 2.2.11 Real-time quantitative PCR

Total RNA was extracted from BEAS-2B cells using the Solarbio kit (R1200) and assessed for quality using a NanoDrop spectrophotometer and agarose gel electrophoresis. cDNA was synthesized with the iScript cDNA Synthesis Kit (Bio-Rad, 1708890), and RT-qPCR was performed on a CFX96 system (10 μL: 2μL cDNA+5μL SYBR Green Supermix (Bio-Rad, 1708880)+0.5μL each primer+2μL nuclease-free water). The cycling conditions were: 95 °C for 30 s, followed by 40 cycles of 95 °C for 10 s and 60 °C for 30 s. Gene expression was analyzed using the 2^^(-ΔΔCt)^ method with GAPDH as the internal reference. The sequences of primers used in this study are listed in **Table 1**.

**Table 1:** List of primers for RT-qPCR analysis.

Gene	Oligonucleotide sequence	
ELAVL1	Forward	5′-CATGACCCAGGATGAGTTACGAAGC-3′
	Reverse	5′-GGCGAGCATACGACACCTTAATGG-3′
SAT2	Forward	5′-CCGCTCTCTCAGGCTCTTCAGG-3′
	Reverse	5′-CATCTGCTCTCAGGGCTTCTTCAC-3′
RUNX1	Forward	5′-TCTTCACAAACCCACCGCAA-3′
	Reverse	5′-CTGCCGATGTCTTCGAGGTTC-3′
ALDH1A1	Forward	5′-ACGCCAGACTTACCTGTCCTACTC-3′
	Reverse	5′-CTCCATTGTCGCCAGCAGCAG-3′
SLC7A11	Forward	5′-TCCGCAAGCACACTCCTCTACC-3′
	Reverse	5′-GTGATGACGAAGCCAATCCCTGTAC-3′
GAPDH	Forward	5′-GGAGCGAGATCCCTCCAAAAT-3′
	Reverse	5′-GGCTGTTGTCATACTTCTCATGG-3′

#### 2.2.12 Molecular docking and binding mode prediction of RUNX1-farrerol complex

The full-length structure of human RUNX1 (UniprotKB: Q01196) was predicted using I-TASSER (https://zhanggroup.org/I-TASSER/). The V54-R177 region was modeled based on the crystal structure (PDB: 1H9D), followed by energy minimization with MOE (Molecular Operating Environment, Chemical Computing Group ULC. v2022.02) software to obtain a stable conformation. Docking of farrerol with RUNX1 was performed using AutoDock-GPU (accelerated version of AutoDock4.2.6). The predicted RUNX1 structure served as receptor, while farrerol structure was obtained from PubChem (https://pubchem.ncbi.nlm.nih.gov/). The Lamarckian Genetic Algorithm and empirical free energy scoring function were employed, with the binding pocket predicted by CavityPlus (http://www.pkumdl.cn:8000/cavityplus/#/computation). The grid was centered at 80.25, 116.5, 137.0 Å (30×30×30 Å). After 100 genetic algorithm runs, the conformation with optimal docking score was selected. Molecular interactions were analyzed using Discovery Studio Visualizer (v18.1), and 3D models were generated with PyMol (v2.5.0).

#### 2.2.13 Lipid peroxidation assay

Intracellular lipid peroxidation was assessed via flow cytometry using the BODIPY 581/591 C11 Lipid Peroxidation Assay Kit (Beyotime Biotechnology, Shanghai, China, S0043S), following the manufacturer’s instructions. Briefly, treated BEAS-2B cells were digested into single−cell suspensions, incubated with 2 µM C11 BODIPY dye in serum-free medium at 37 °C in the dark for 20 min, washed with PBS, and analyzed immediately using a flow cytometer.

#### 2.2.14 Mitochondrial transmission electron microscopy

To examine mitochondrial ultrastructural alterations in BEAS-2B cells, TEM analysis was conducted. Following treatment, cells were immediately fixed in ice-cold 2.5% glutaraldehyde, then processed through dehydration, embedding, and staining. Samples were analyzed by TEM (Lilai Biotechnology Co., Ltd.) to assess mitochondrial morphology, including cristae structure.

#### 2.2.15 Malondialdehyde and glutathione analysis

Intracellular levels of GSH and MDA were quantitatively determined in BEAS-2B using commercial assay kits (Beyotime Biotechnology, Shanghai, China), following the manufacturer’s standardized protocols.

### 2.3 *In vivo* experiments

#### 2.3.1 Animal care and welfare

A total of 70 healthy male C57BL/6 mice (6–8 weeks old, 25–30 g; purchased from Daqo Experimental Animal Co., Ltd.) were used in this study. All animals were housed under specific pathogen-free (SPF) conditions with controlled temperature (22 ± 2 °C), humidity (55 ± 10%), and a 12-hour light/dark cycle, with free access to food and water. All experimental procedures were approved by the Animal Ethics Committee of Chengdu Medical College (Approval No. CMC-IACUC-2021024) and conducted in strict accordance with the 3R principles.

#### 2.3.2 Establishment of ALI mouse model

A total of 50 C57BL/6 mice were used to establish an ALI model and randomly divided into the following groups: Control group (0.9% NaCl + 0.1% DMSO), LPS group (LPS dissolved in 0.9% NaCl containing 0.1% DMSO), and LPS + Farrerol groups (10, 20, or 40 mg/kg, administered via tail vein injection 1 hour prior to LPS exposure). ALI was induced by intratracheal instillation of LPS (5 mg/kg). The LPS dosing regimen followed that reported in Zhou et al. (24). An additional 20 mice were divided into four groups (n = 5 per group) to investigate the mechanism of ferroptosis. Mice were fasted for 12 h before the experiment, anesthetized, and administered 10 mg/kg LPS via intratracheal instillation. Lung tissues and bronchoalveolar lavage fluid (BALF) were collected 12 h post-LPS administration.

#### 2.3.3 Evans blue staining

After LPS treatment of mice via intratracheal instillation (5 mg/kg) for 4 h, Evans blue dye (50 mg/kg) was administered via tail vein injection. After 2 h, mice were anesthetized and perfused with saline through the right ventricle for 5 min.

#### 2.3.4 MDA and GSH analysis

GSH and MDA levels in lung tissue were measured using a commercial kit ((Beyotime Biotechnology, Shanghai, China), according to the manufacturer’s instructions.

#### 2.3.5 Wet-to-dry ratio (W/D ratio) measurement, hematoxylin and eosin staining, TUNEL assay, and immunohistochemical analysis of mouse lung tissue

To assess the protective effects of farrerol, we evaluated pulmonary edema based on the W/D ratio, histopathological alterations via H staining, cellular apoptosis using TUNEL assays, and SLC7A11 and 4-HNE protein expression through immunohistochemistry (IHC). All experimental procedures were performed following our previously established protocols (12).

### 2.4 Statistical analysis

Data from this study are expressed as the mean ± standard deviation (mean ± SD). Statistical analysis was conducted utilizing GraphPad Prism 9.0 software (GraphPad Software, Inc., La Jolla, CA, USA). To compare multiple groups, a one-way analysis of variance (ANOVA) was employed, followed by Tukey’s *post hoc* test for pairwise comparisons. For direct comparisons between the two groups, an independent samples t-test was applied. The threshold for statistical significance was set at p < 0.05. Each experiment was conducted in triplicate or more to ensure the reliability and statistical robustness of the findings.

## 3 Results

### 3.1 Farrerol inhibited LPS-induced injury in BEAS-2B cells

**Figure 1:**
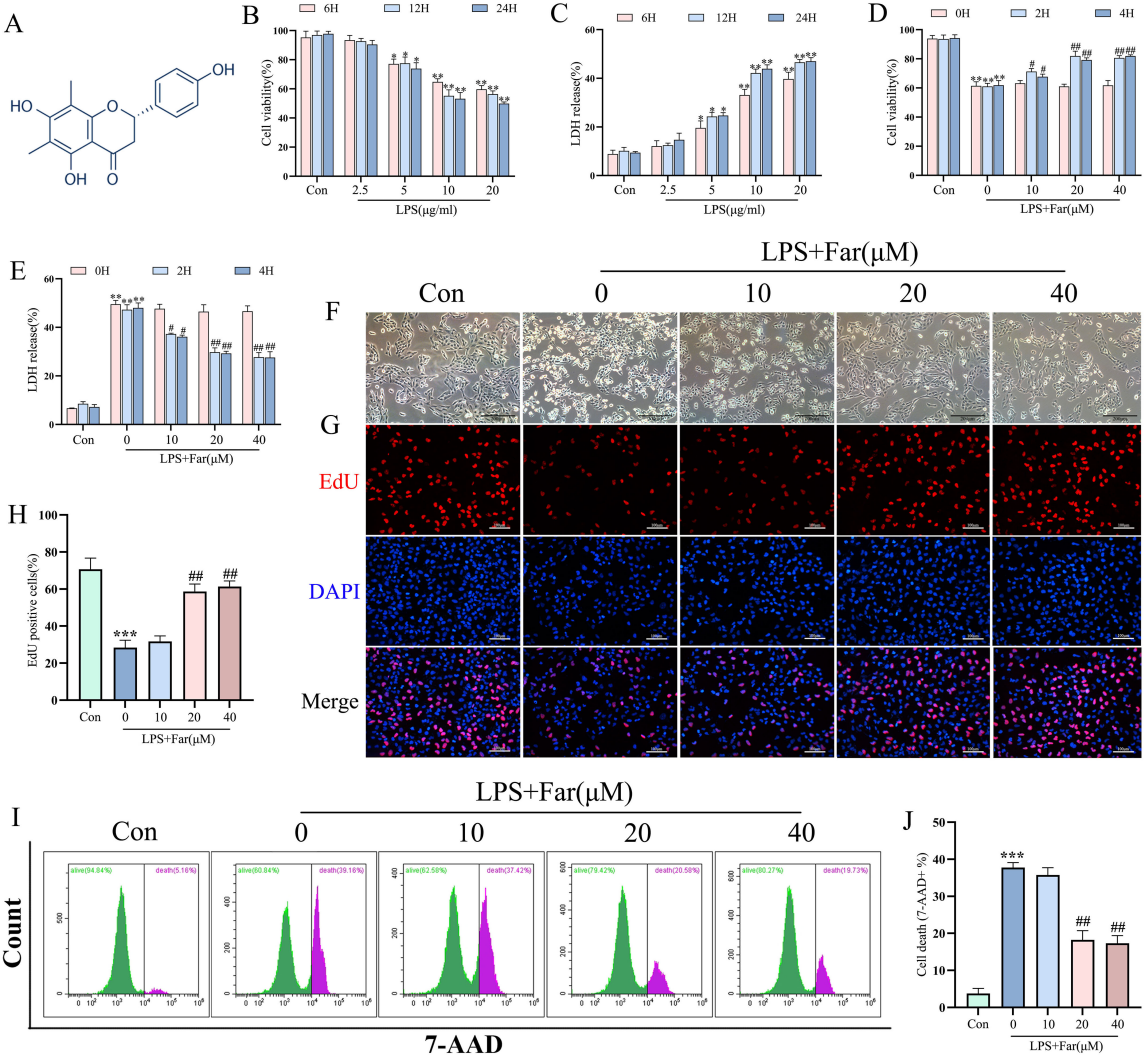
Evaluation of the protective effect of farrerol on LPS-induced BEAS-2B cell injury. **(A)** Molecular structure of Farrerol. **(B, C)** Cell viability and injury in BEAS-2B cells treated with varying concentrations of LPS (2.5, 5, 10, and 20 μg/mL) for different durations (6, 12, and 24 hours), assessed using the CCK-8 and LDH release assay. **(D, E)** Cell viability and injury in BEAS-2B cells pretreated with varying concentrations of Farrerol (10, 20, and 40 μM) for different durations (0, 2, and 4 hours), evaluated using the CCK-8 and LDH release assay. **(F)** Representative phase-contrast microscopy images showing the morphology of BEAS-2B cells in each group. Scale bar: 200 μm. **(G, H)** Representative fluorescence images from the EDU assay. Scale bar: 100 μm. **(I, J)** Representative 7-AAD flow cytometry images. All data are presented as mean ± SEM (n=3 for each group). *P < 0.05, **P < 0.01,***P < 0.001 vs. control; #P < 0.05, ##P < 0.01 vs. LPS.

To evaluate the effects of farrerol on LPS-induced injury *in vitro*, we established a BEAS-2B cell damage model. The chemical structure of farrerol is illustrated in **Figure 1A**. The cell counting kit-8 and LDH release assays demonstrated that LPS treatment decreased cell viability and increased LDH release in a dose- and time-dependent manner (**Figures 1B, C**). Based on these observations, treatment with 10 µg/mL LPS for 12 h was selected as the standardized injury model for subsequent experiments due to its optimal cytotoxic effects. Optimization of farrerol pretreatment at different concentrations (10, 20, and 40 μM) and time points (0, 2, and 4 h) revealed a dose-dependent improvement in cell viability and reduction in LDH release (**Figures 1D, E**), preservation of normal cell morphology (**Figure 1F**), enhanced cell proliferation (**Figures 1G, H**), and decreased cell death (**Figures 1I, J**). Pretreatment with 20 μM farrerol for 2 h produced the most significant protective effect. Taken together, these results suggest that farrerol exerts protective effects against LPS-induced BEAS-2B injury.

### 3.2 Farrerol alleviated LPS-induced acute lung injury in mice

**Figure 2:**
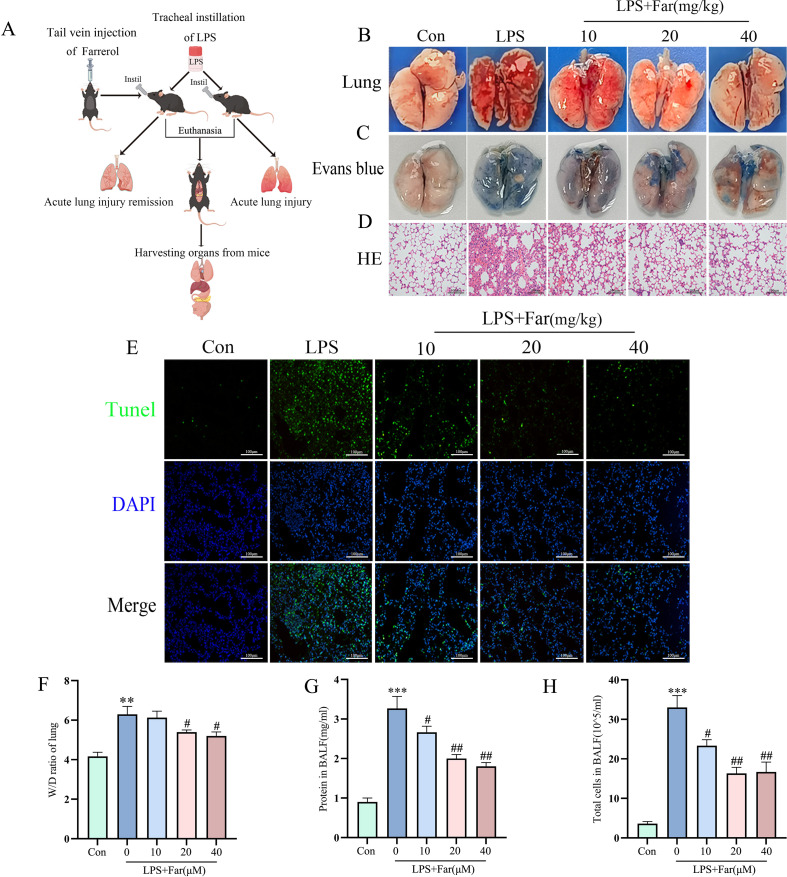
Farrerol treatment alleviates pathological injury and inflammatory responses in LPS-induced ALI. **(A)** Schematic representation of the animal experimental design. **(B)** Representative images of mouse lung tissues (n=4). **(C)** Representative images of Evans Blue staining (n=3). **(D)** Representative images of hematoxylin and eosin (HE) staining. Scale bar: 100 μm. **(E)** Representative fluorescence images of TUNEL staining. Scale bar: 100 μm. **(F)** Lung edema assessed by the wet-to-dry (W/D) weight ratio(n=3). **(G, H)** Total protein concentration and total cell count in BALF. All data are presented as mean ± SEM (n=10 for each group). **P < 0.01, ***P < 0.001 vs. control; #P < 0.05, ##P < 0.01 vs. LPS.

Using the LPS-induced ALI model (**Figure 2A**), we found that pretreatment with farrerol (10, 20, and 40 mg/kg) alleviated pathological lung tissue damage in a dose-dependent manner (**Figures 2B, D**). Specifically, Evans blue staining demonstrated a marked attenuation of LPS-induced pulmonary vascular leakage following farrerol treatment (**Figure 2C**). Additionally, the TUNEL assay showed significant inhibition of LPS-induced cell death in the lung tissue. Furthermore, farrerol administration notably decreased the wet/dry weight ratio, protein concentration, and total cell count in the bronchoalveolar lavage fluid of LPS-exposed mice (**Figures 2F–H**). Collectively, these data suggest that farrerol alleviates LPS-induced ALI in mice.

### 3.3 Farrerol directly binds to and upregulates RUNX1

**Figure 3:**
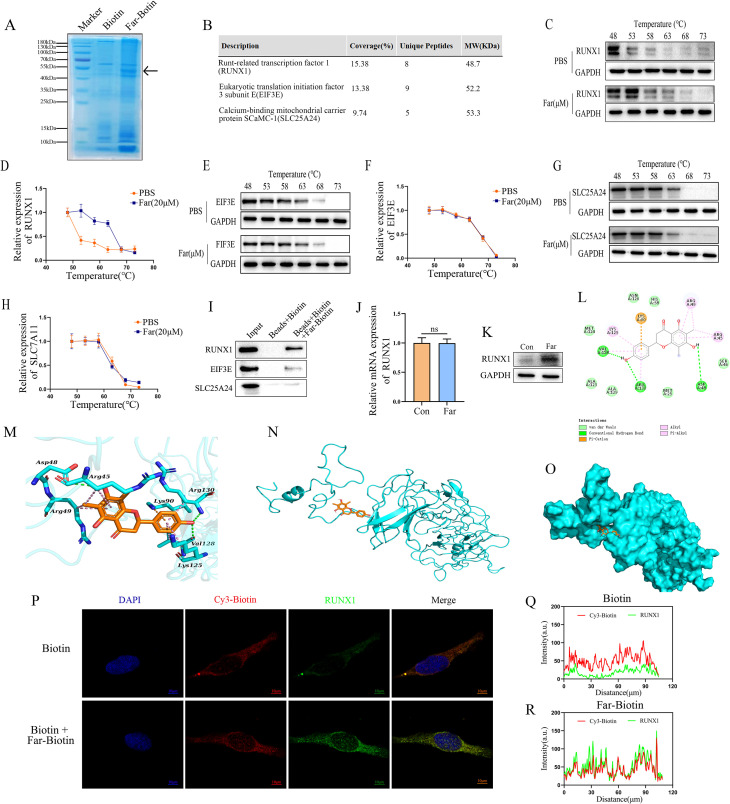
Identification of potential binding targets of farrerol in BEAS-2B cells. **(A)** Coomassie brilliant blue staining. **(B)** Proteomic analysis identified three potential Farrerol targets: RUNX1, EIF3E, and SLC25A24. **(C, D)** CETSA and Western blot analysis of the interaction between Farrerol and RUNX1. **(E, F)** CETSA and Western blot analysis of the interaction between Farrerol and EIF3E. **(G, H)** CETSA and Western blot analysis of the interaction between Farrerol and SLC25A24. **(I)** Co-IP analysis was performed to evaluate the interaction between RUNX1, SLC25A24, EIF3E, and Far-Biotin. The lanes from left to right are: Input (total cell lysate), Beads +Biotin (negative control), and Beads+Biotin+Far-Biotin (experimental group). **(J, K)** After 24 hours of farrerol treatment, the mRNA and protein expression levels of RUNX1 were determined by qPCR and Western blot analysis, respectively. **(L)** The 2D binding mode of farrerol and RUNX1. **(M–O)** The 3D binding mode of farrerol and RUNX1. The farrerol was colored in orange. The backbone of RUNX1 was shown as cyan surface and cartoon. The residues in the binding pocket of human RUNX1 were shown as cyan sticks. The conventional hydrogen bond interactions were depicted as green dashed lines. The alkyl-alkyl hydrophobic interactions and pi-alkyl hydrophobic interactions were depicted as pink dashed lines. The pi-cation electrostatic interactions were depicted as orange dashed lines. **(N–R)** Cellular immunofluorescence colocalization of Biotin-Far and RUNX1. Scale bar: 10 μm. All data are presented as mean ± SEM (n=3 for each group).

We further investigated the direct molecular targets of farrerol in BEAS-2B cells. Pull-down assays coupled with mass spectrometry identified three candidate target proteins: RUNX1, EIF3E, and SLC25A24 (**Figures 3A, B**). CETSA analysis revealed that farrerol significantly enhanced the thermal stability of RUNX1 (**Figures 3C–H**), and co-immunoprecipitation confirmed their specific interactions (**Figure 3I**). Immunofluorescence staining showed enhanced nuclear co-localization of farrerol and RUNX1 (**Figures 3P–R**). Molecular docking further revealed that farrerol binds to the DNA-binding domain of RUNX1 via four hydrogen bonds (Arg45, Asp48, Lys90, and Arg130) (**Figures 3L–O**). In addition, farrerol upregulated RUNX1 protein expression in a dose-dependent manner, without affecting its mRNA levels (**Figures 3, K**). Collectively, these results indicate that farrerol exerts its protective effects by directly binding to and upregulating RUNX1 expression.

### 3.4 RUNX1 overexpression significantly inhibits ferroptosis

**Figure 4:**
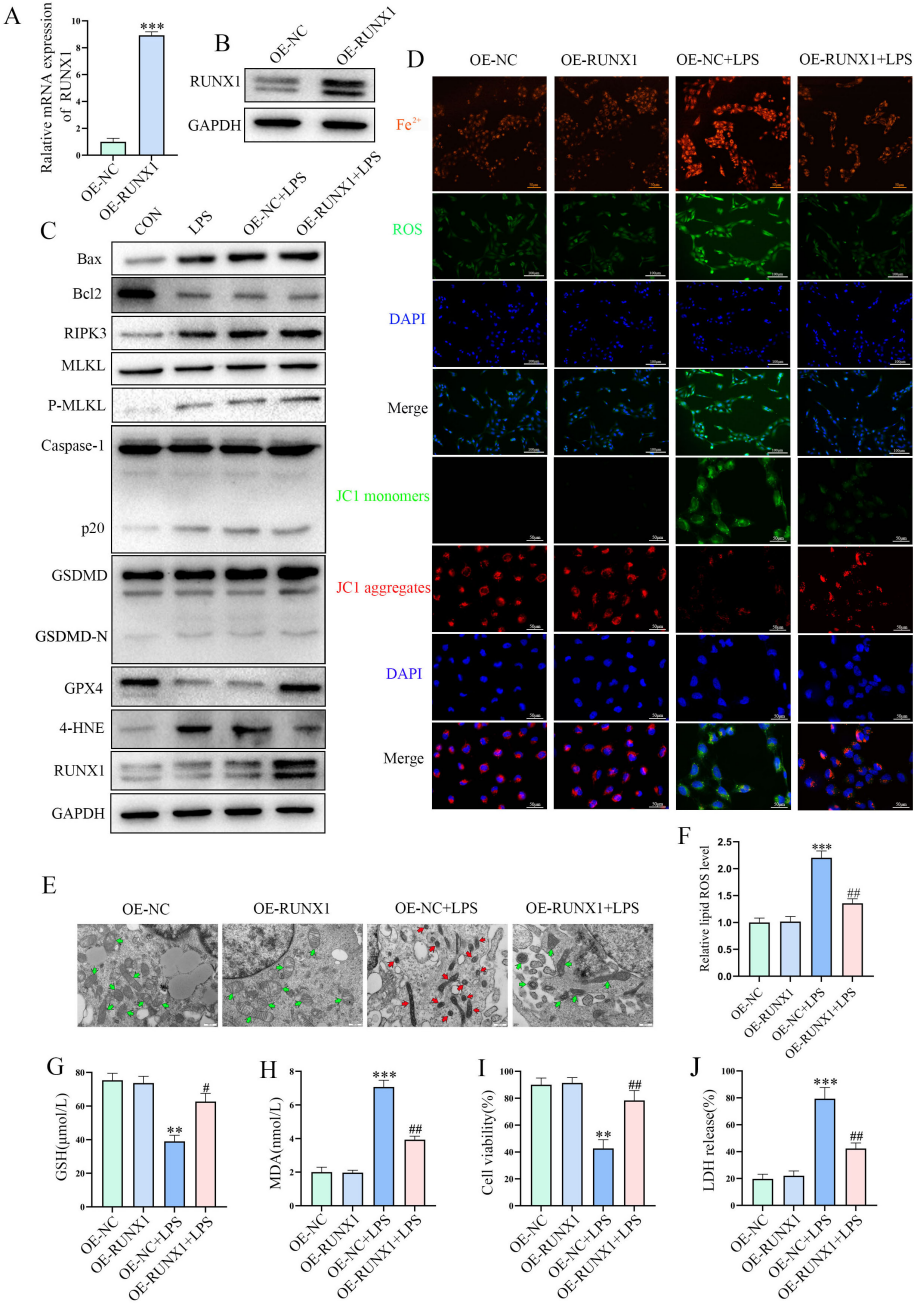
RUNX1 regulates cell death pathways and farrerol’s protective mechanism. **(A, B)** RT-qPCR and Western blot analysis of RUNX1 overexpression efficiency in BEAS-2B cells. **(C)** Western blot analysis of the effects of RUNX1 overexpression on different cell death pathways following LPS treatment. **(D)** Detection of Fe^2+^ levels, ROS production, and JC-1 fluorescence. Scale bars: 50 μm and 100 μm. **(E)** Transmission electron microscopy showing ultrastructural changes in mitochondria across groups. The red arrows indicate mitochondria with damaged morphology, the green arrows represent mitochondria with normal morphology. Scale bar: 500 nm. **(F)** Detection of intracellular lipid peroxidation levels (BODIPY 581/591C11 Method). **(G, H)** GSH and MDA levels were measured. **(I, J)** CCK-8 and LDH assays. All data are presented as mean ± SEM (n=3 for each group). **P < 0.01, ***P < 0.001 vs. OE-NC; #P < 0.05, ##P < 0.01, vs. OE-NC + LPS.

Following prior identification of a direct farrerol–RUNX1 interaction, RUNX1 was overexpressed in BEAS-2B cells to further elucidate its functional role (**Figures 4A, B**). This allowed investigation of its potential involvement in regulating programmed cell death pathways, including apoptosis, necroptosis, pyroptosis, and ferroptosis (**Figure 4C**). RUNX1 overexpression specifically modulated ferroptosis-related markers, notably GPX4 and 4-HNE (**Figure 4C**), and reversed LPS-induced Fe^2+^ accumulation, ROS burst, mitochondrial damage, and lipid peroxidation (**Figures 4D–F**). Quantitative analyses further confirmed that RUNX1 maintained GSH levels, reduced MDA production, enhanced cell viability, and decreased LDH release (**Figures 4F–I**), thus fully recapitulating the protective effects of farrerol. These findings highlight the “farrerol–RUNX1–ferroptosis inhibition” axis as a key mechanism underlying pulmonary protection.

### 3.5 Farrerol protects against erastin-induced ferroptosis *in vitro*

To further validate the protective effect of farrerol in a classic ferroptosis model independent of LPS, the ferroptosis inducer erastin was employed. BEAS−2B cells were pretreated with farrerol (20 μM for 2 h) followed by exposure to erastin (1 μM) for 24 h (25). As shown in **Supplementary Figure 1**, erastin significantly reduced cell viability, an effect that was effectively reversed by co−treatment with farrerol (**Supplementary Figure 1A**). Consistently, farrerol markedly attenuated erastin−induced lipid peroxidation, as indicated by decreased oxidation of the C11−BODIPY probe (**Supplementary Figure 1B**), and reversed the depletion of GSH and accumulation of MDA (**Supplementary Figures 1C, D**). Furthermore, farrerol suppressed intracellular Fe^2+^ overload (**Supplementary Figure 1E**) and alleviated erastin−induced ultrastructural mitochondrial damage, including mitochondrial shrinkage and cristae disruption (**Supplementary Figure 1F**). These results demonstrate that farrerol exerts broad protective effects against ferroptosis triggered by diverse inducers.

### 3.6 RUNX1 knockdown reverses the farrerol−induced inhibition of ferroptosis *in vitro*

**Figure 5:**
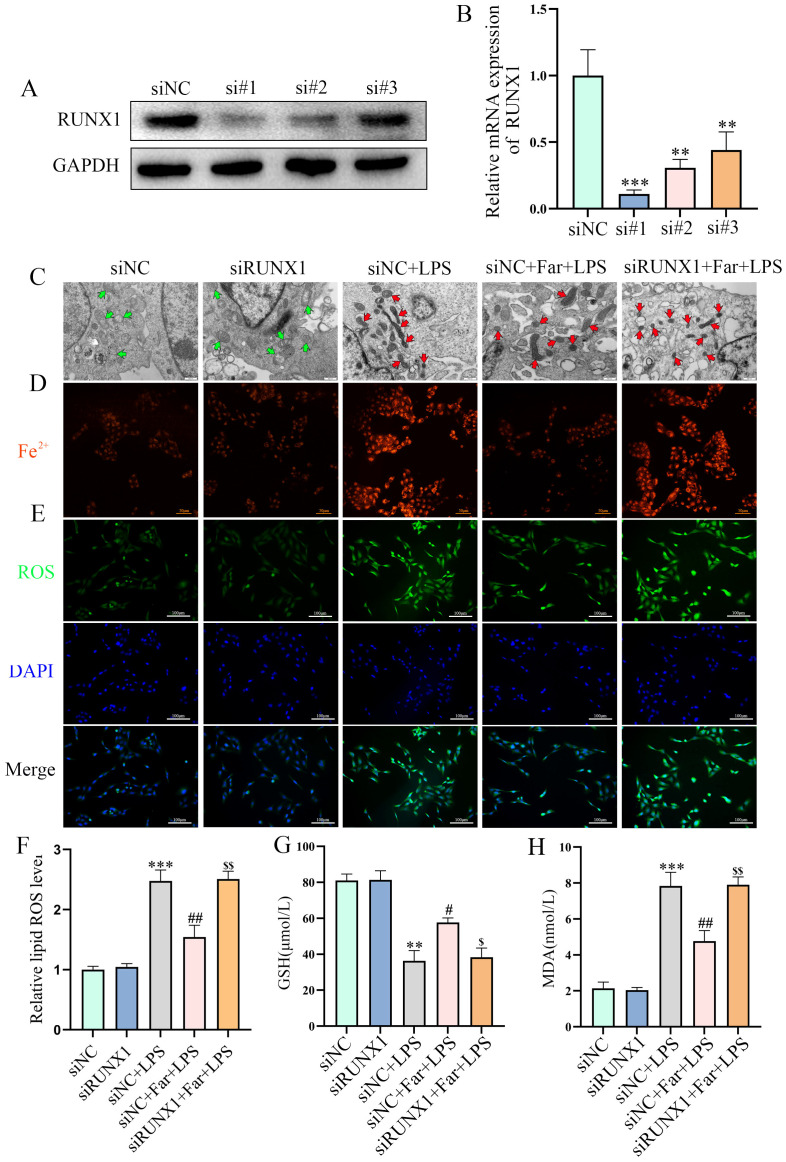
Role of RUNX1 in farrerol regulation of ferroptosis. **(A, B)** Western blot and RT-qPCR analysis of RUNX1 knockdown efficiency in BEAS-2B cells. **(C)** Transmission electron microscopy showing ultrastructural changes in mitochondria across groups. The red arrows indicate mitochondria with damaged morphology, the green arrows represent mitochondria with normal morphology. **(D, E)** Fe^2+^ and ROS detection. Scale bars: 50 μm and 100 μm. **(F)** Detection of intracellular lipid peroxidation levels (BODIPY 581/591C11 Method). **(G, H)** GSH and MDA levels were measured. All data are presented as mean ± SEM (n=3 for each group). **P < 0.01, ***P < 0.001vs. siNC group, #P< 0.05, ##P < 0.01, vs siNC+LPS, $P< 0.05, $$P < 0.01, vs siNC+Far+LPS.

To further verify the role of RUNX1 in farrerol−induced inhibition of ferroptosis, we downregulated RUNX1 expression in BEAS-2B cells. RUNX1 knockdown (**Figures 5A, B**) significantly abolished the protective effects of farrerol on LPS-induced ferroptotic features, including mitochondrial cristae disruption and membrane rupture (**Figure 5C**), increased Fe^2+^ accumulation (**Figure 5D**, enhanced reactive oxygen species (ROS) production (**Figure 5E**), and elevated lipid peroxidation (**Figure 5F**), as well as imbalances in GSH and MDA levels (**Figures 5F, G**). Collectively, these findings demonstrate that the inhibitory effect of farrerol on LPS-induced ferroptosis is mediated by RUNX1.

### 3.7 RUNX1 suppresses ferroptosis by promoting the transcription of *SLC7A11*

**Figure 6:**
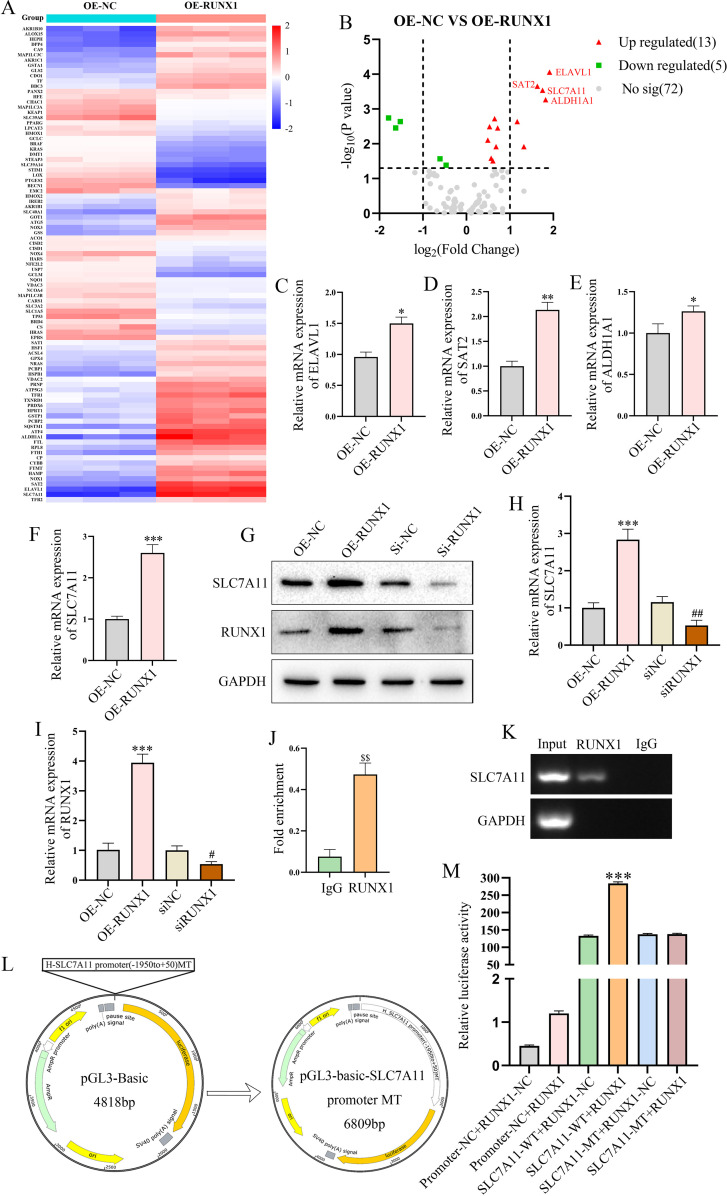
RUNX1-regulated SLC7A11 expression as a key mechanism in ferroptosis regulation. **(A)** Heatmap of ferroptosis-related gene expression from the PCR array. **(B)** Volcano plot of upregulated and downregulated genes. **(C-F)** qPCR validation of RUNX1 effects on ferroptosis significantly upregulated genes ELAVL1, SAT2, ALDH1A1, and SLC7A11. **(G)** Western blot analysis of the effects of RUNX1 overexpression or knockdown on SLC7A11 protein expression. **(H-I)** qPCR analysis of SLC7A11 and RUNX1 mRNA expression levels. **(J)** Results of ChIP-qPCR experiments. **(K)** Agarose gel electrophoresis results. **(L)** Schematic diagram of the SLC7A11 promoter mutant plasmid (SLC7A11-Mut) construction. **(M)** Dual-luciferase reporter assay results. All data are presented as mean ± SEM(n=3 for each group). *P < 0.05, **P < 0.01, ***P < 0.001 vs. OE-NC or Promoter-NC + RUNX1-NC; #P < 0.05, ##P < 0.01 vs. siNC group;$$P < 0.01 vs. IgG.

To identify the downstream targets through which RUNX1 regulates ferroptosis, we performed a ferroptosis-related gene PCR array and found that RUNX1 overexpression markedly altered the expression profiles of multiple ferroptosis-associated genes (**Figures 6A, B**). Among the 13 upregulated genes, we selected the four that were most significantly upregulated (*ELAVL1*, *SAT2*, *ALDH1A1*, and *SLC7A11*) for qPCR validation, and identified *SLC7A11* as the most prominently upregulated gene in response to RUNX1 overexpression. (**Figures 6C–F**). Bidirectional validation further confirmed that RUNX1 overexpression upregulated, whereas RUNX1 knockdown suppressed *SLC7A11* expression at both the mRNA and protein levels (**Figures 6G–I**). Chromatin immunoprecipitation and dual-luciferase reporter assays demonstrated that RUNX1 directly binds to and transcriptionally activates the *SLC7A11* promoter, with site-specific binding confirmed via mutational analysis (**Figures 6J–M**). These results indicate that *SLC7A11* is a key effector mediating the anti-ferroptotic function of RUNX1.

### 3.8 The protective role of farrerol in alleviating LPS-induced ferroptosis *in vivo*

**Figure 7:**
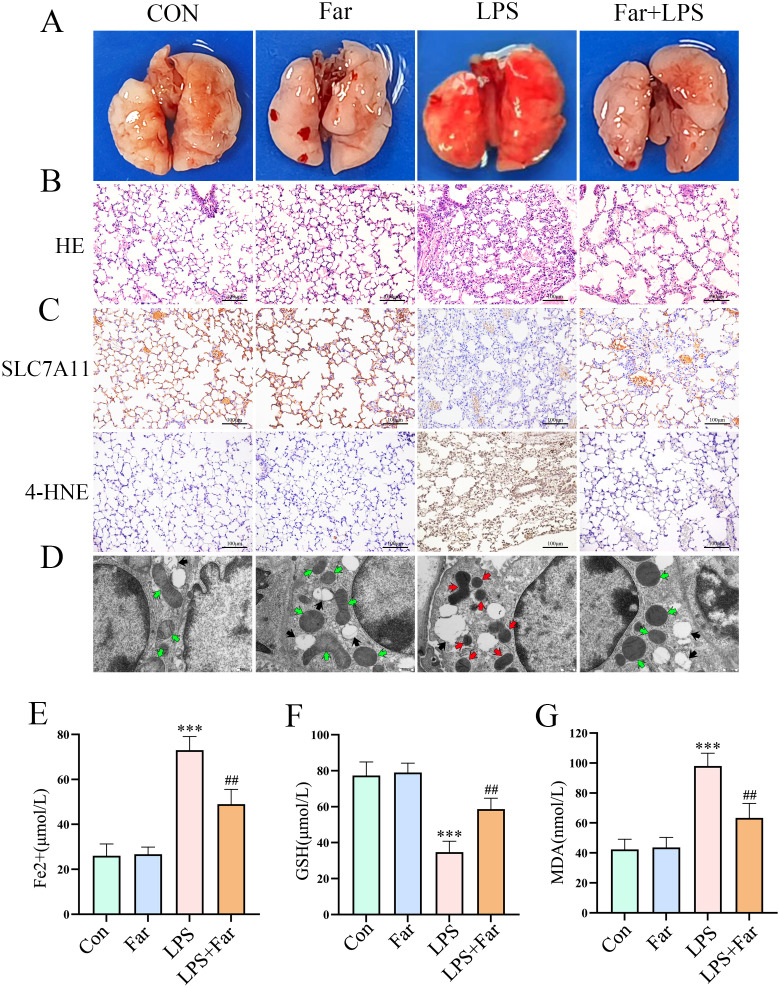
The protective role of farrerol in alleviating LPS-Induced ferroptosis *in vivo*. **(A)** Representative images of mouse lung tissues (n=5). **(B)** Representative images of hematoxylin and eosin (HE) staining. Scale bar:100μm. **(C)** Representative immunohistochemical images of SLC7A11 and 4-HNE staining. Scale bar: 100 μm. **(D)** Transmission electron microscopy showing ultrastructural changes in mitochondria in type II alveolar epithelial cells. The red arrows indicate mitochondria with damaged morphology, the green arrows represent mitochondria with normal morphology, and the black arrows denote alveolar type II epithelial cell-specific lamellar bodies. Scale bar: 500 nm. **(E–G)** Measurements of Fe^2+^ levels, GSH, and MDA in lung tissues. All data are presented as mean ± SEM. ***P < 0.001 vs. control; ##P < 0.01 vs. LPS.

To determine whether farrerol suppresses ferroptosis *in vivo*, ferroptosis-related parameters were systematically assessed in an LPS-induced ALI mouse model. Histopathological analysis revealed that farrerol pretreatment alleviated pulmonary congestion, edema, and alveolar destruction (**Figures 7A, B**). Moreover, farrerol reversed LPS-induced downregulation of *SLC7A11* and upregulation of 4-HNE (**Figure 7C**). TEM analysis further confirmed that farrerol preserved mitochondrial integrity, in contrast to the disrupted cristae and damaged membranes observed in the LPS group (**Figure 7D**). Biochemical assays demonstrated that farrerol pretreatment reduced iron and MDA levels while restoring GSH content in lung tissues (**Figures 7E–G**). These findings suggest that farrerol exerts protective effects against lung injury by inhibiting ferroptosis.

### 3.9 Potential toxicity and safety evaluation of farrerol

**Figure 8:**
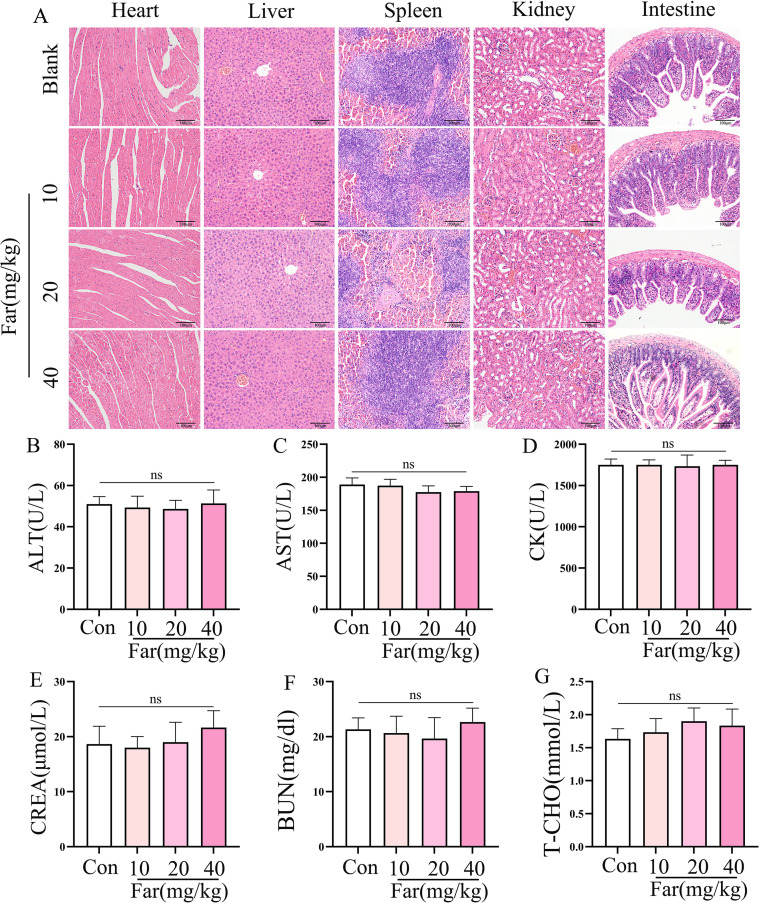
Potential toxicity and safety evaluation of farrerol. **(A)** Representative hematoxylin and eosin (HE) staining images of mouse heart, liver, spleen, kidney, and intestine tissues after Farrerol treatment (n = 6 per group). Scale bar: 100 μm. **(B–G)** Biochemical measurements of serum AST, ALT, CK, CREA, BUN, and T-CHO levels in mice. All data are presented as mean ± SEM.

Building on the confirmed protective effects of farrerol against ALI, we evaluated it’s *in vivo* toxicity profile. Farrerol administration at doses of 10, 20, and 40 mg/kg did not induce pathological damage in major organs (heart, liver, spleen, kidney, and intestine; **Figure 8A**). Moreover, it did not cause significant alterations in key serum biochemical parameters, including alanine aminotransferase, aspartate aminotransferase, creatinine, blood urea nitrogen, creatine kinase, and total cholesterol (**Figures 8B–G**). These results demonstrate that farrerol possesses a favorable therapeutic safety window, providing critical preclinical evidence to support its potential for clinical translation.

## 4 Discussion

This study systematically elucidates how farrerol inhibits ferroptosis by targeting the RUNX1–*SLC7A11* signaling axis, thereby mitigating LPS-induced ALI. Using both *in vitro* and *in vivo* models, we demonstrated that farrerol stabilizes the protein expression of the transcription factor RUNX1. This in turn activates the transcriptional activity of the downstream antioxidant gene *SLC7A11*, leading to the inhibition of lipid peroxidation and disruption of iron homeostasis. This mechanism provides new insights into the potential therapeutic strategies for ALI.

Farrerol, an active compound isolated from natural rhododendron plants, has been shown to mitigate inflammatory and oxidative damage in various pathological contexts. For instance, it inhibited β-amyloid-induced neuroinflammation via the Nrf2/Keap1 pathway (17), alleviated high glucose-induced renal mesangial cell injury through the ROS/Nox4/ERK1/2 axis (16), and reduced cerebral ischemia-reperfusion injury by promoting neuronal survival and suppressing neuroinflammation (26). ALI is characterized by intense inflammatory and oxidative damage; however, whether farrerol also exerts protective effects in ALI remains unclear. Our *in vitro* experiments demonstrated that pretreatment with farrerol significantly ameliorated LPS-induced injury in BEAS-2B cells, as evidenced by restored cell viability, reduced LDH release, and suppressed cell death. Correspondingly, in an LPS-induced mouse model of ALI, farrerol pretreatment effectively alleviated alveolar destruction and vascular leakage. Furthermore, within the dose range employed in this study, farrerol did not exhibit significant organ toxicity nor did it induce abnormalities in serum biochemical parameters. These findings confirm the lung-protective effects of farrerol and highlight its favorable safety and efficacy profile. However, a previous study reported no significant protective effect of farrerol in an LPS-induced ALI model (27). This discrepancy may be attributed to several factors, including differences in mouse strain and sex, which can influence drug absorption and efficacy. Variations in the route of administration, dosage, and timing of farrerol intervention may also affect its bioavailability and distribution in lung tissue. Further research is warranted to elucidate the underlying mechanistic differences.

In recent years, studies have indicated that ferroptosis is involved in ALI and that inhibiting ferroptosis can effectively alleviate ALI (28, 29). Previous research has shown that farrerol can inhibit ferroptosis. For example, farrerol has been reported to protect against glaucoma neuropathy and hypoxic-ischemic encephalopathy by activating the Nrf2 antioxidant pathway to counteract ferroptosis (30, 31). Additionally, farrerol alleviated tendinopathy by antagonizing GPX4 inhibition (21). Our results indicate that besides inhibiting LPS-induced ferroptosis, farrerol also exhibits anti-ferroptotic effects in a classic erastin-induced model. This discovery aligns with and extends previous reports on the protective role of farrerol in ferroptosis-related diseases such as tendinopathy and hypoxic-ischemic encephalopathy. Using proteomics, CETSA, Co-IP, and molecular docking approaches, we confirm that farrerol directly targets RUNX1. RUNX1 is a member of the RUNX family of transcription factors and plays a crucial role in various biological processes by regulating multiple cellular functions in mammals. Previous studies suggested that the RUNX1 intronic transcript (RUNX1-IT1) can inhibit ferroptosis in breast cancer cells by stabilizing GPX4 mRNA (32). In this study, we found that RUNX1 specifically inhibited ferroptosis in pulmonary epithelial cells, rather than other forms of cell death such as apoptosis, necroptosis, or pyroptosis. Furthermore, based on a ferroptosis-focused PCR array and chromatin immunoprecipitation assays, we demonstrated that RUNX1 binds to the SLC7A11 promoter, enhances its transcriptional activity, and upregulates SLC7A11 expression, thereby promoting cystine uptake and GSH synthesis, and ultimately inhibiting ferroptosis. Compared with previous studies, our work reveals a novel mechanism by which farrerol inhibits ferroptosis.

We reveal a novel mechanism by which farrerol inhibits ferroptosis through the RUNX1/SLC7A11 axis, providing a new potential therapeutic target for ALI. Our work identifies RUNX1 as a novel transcriptional regulator of ferroptosis in pulmonary epithelial cells, elucidates its unique protective mechanism in ALI, and expands our understanding of the functions of the transcription factor RUNX1. However, our study also has several limitations. First, although molecular docking was employed to predict the binding sites between RUNX1 and farrerol, these predictions were not experimentally validated. Structure-based drug design critically depends on precise understanding of binding site conformation and interactions (33). Therefore, structural biological studies of the RUNX1-farrerol complex represent an important direction for future research. Second, the pathological process of ALI involves injury to multiple cell types, including bronchial epithelial, alveolar epithelial, and pulmonary vascular endothelial cells (34, 35). While the present study primarily focused on the protective effect of farrerol on bronchial epithelial cells, its impact on other pulmonary cell types (e.g., alveolar epithelial and endothelial cells) remains unclear, which provides a new direction for subsequent investigations. Finally, both cecal ligation and puncture (CLP) and LPS stimulation are classic models of ALI (36). However, only the LPS model was employed in the current study; thus, our findings may require further validation in CLP-induced ALI models in the future.

## 5 Conclusion

**Figure 9:**
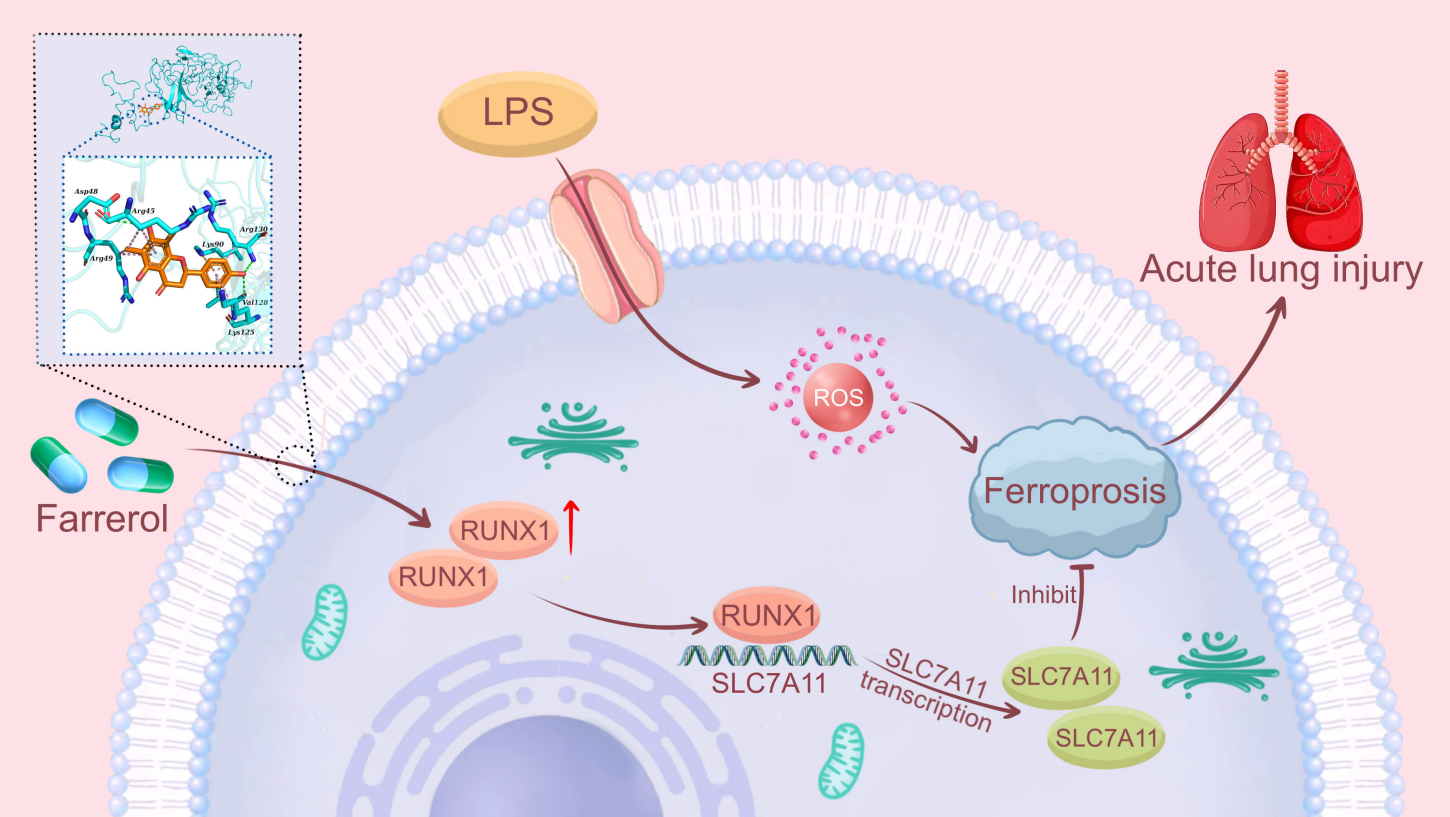
Farrerol alleviates LPS-induced acute lung injury by regulating the UCHL3/RUNX1/SLC7A11 axis to inhibit ferroptosis. Schematic representation of the mechanism by which LPS stimulation induces ROS accumulation, ferroptosis, and acute lung injury (ALI). Farrerol pretreatment activates UCHL3, which binds to RUNX1, preventing its ubiquitin-mediated degradation and stabilizing RUNX1 protein levels. Stabilized RUNX1 binds to the SLC7A11 promoter region, promoting SLC7A11 transcription. Upregulation of SLC7A11 inhibits ferroptosis, thereby alleviating LPS-induced ALI.

This study demonstrates that farrerol significantly alleviates LPS-induced ALI, primarily by inhibiting ferroptosis. Mechanistically, farrerol directly binds to and stabilizes the transcription factor RUNX1, thereby upregulating the transcriptional activity of its downstream target gene *SLC7A11*. These findings provide the first evidence that farrerol suppresses ferroptosis by modulating the RUNX1/*SLC7A11* signaling pathway, offering a novel potential therapeutic target for ALI treatment (**Figure 9**).

## Data Availability

The original contributions presented in the study are included in the article/**Supplementary Material**. Further inquiries can be directed to the corresponding author.

## Supplementary material

Supplementary Figure 1 Protective effects of farrerol against erastin−induced ferroptosis in BEAS−2B cells. **(A)** Cell viability (CCK−8 assay) under indicated treatments. **(B)** Detection of intracellular lipid peroxidation levels (BODIPY 581/591C11 Method). **(C, D)** Measurement of intracellular GSH and MDA levels. **(E)** Fluorescence detection of intracellular Fe^2+^ (FerroOrange staining). Scale bar: 50 μm. **(F)** Transmission electron microscopy showing ultrastructural changes in mitochondria across groups. Red arrows indicate mitochondria with damaged morphology, green arrows represent mitochondria with normal morphology. Scale bar: 500 nm. All data are presented as the mean ± SEM (n=3 for each group). ***P < 0.001 vs. Con; ##P < 0.01 vs. erastin.
