# Supplementary figures and images for: Farrerol inhibits ferroptosis and protects against LPS-induced acute lung injury by targeting the RUNX1/SLC7A11 axis
Source: Front Immunol. 2026 Jan 7;16:1720843. doi: 10.3389/fimmu.2025.1720843 (PMC12819189; doi:10.3389/fimmu.2025.1720843)

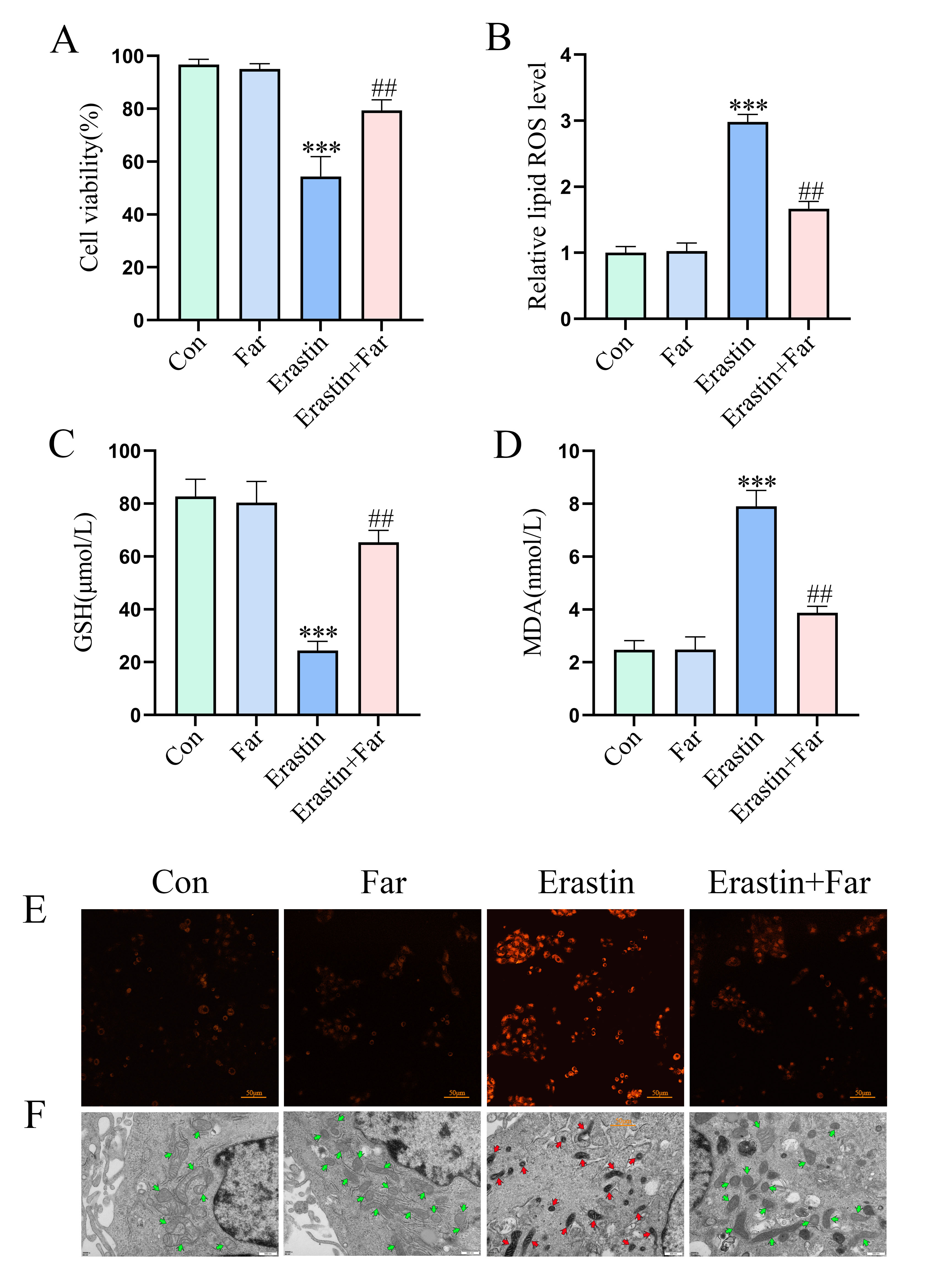

Supplement: Supplementary Figure 1 — Protective effects of farrerol against erastin−induced ferroptosis in BEAS−2B cells. (A) Cell viability (CCK−8 assay) under indicated treatments. (B) Detection of intracellular lipid peroxidation levels (BODIPY 581/591C11 Method). (C, D) Measurement of intracellular GSH and MDA levels. (E) Fluorescence detection of intracellular Fe2+ (FerroOrange staining). Scale bar: 50 μm. (F) Transmission electron microscopy showing ultrastructural changes in mitochondria across groups. Red arrows indicate mitochondria with damaged morphology, green arrows represent mitochondria with normal morphology. Scale bar: 500 nm. All data are presented as the mean ± SEM (n=3 for each group). ***P < 0.001 vs. Con; ##P < 0.01 vs. erastin. [file Image1.jpg]
